# Immunophenotypic shifts during minimal residual evaluation in a case of leukemic form of anaplastic large cell lymphoma ALK+

**DOI:** 10.1002/cnr2.1526

**Published:** 2021-08-11

**Authors:** Maria Clara Canellas, Enrico Bruno‐Riscarolli, Cristiane S. Ferreira‐Facio, Daiana V. Lopes‐Alves, Vitor D. Botafogo, Deborah Sutter, Roberia M. Pontes, Marcelo G. P. Land, Cristiane Bedran Milito, Elaine Sobral da Costa

**Affiliations:** ^1^ Martagão Gesteira Pediatric Institute – Federal University of Rio de Janeiro Rio de Janeiro Brazil; ^2^ Department of Pediatrics Faculty of Medicine ‐ Federal University of Rio de Janeiro Rio de Janeiro Brazil; ^3^ Department of Pathology, Faculty of Medicine Federal University of Rio de Janeiro Rio de Janeiro Brazil; ^4^ Integrated Morphology Laboratory Federal University of Rio de Janeiro Rio de Janeiro Brazil

**Keywords:** anaplastic large cell lymphoma, minimal residual disease, multiparameter flow cytometry

## Abstract

**Background:**

This study aims to describe immunophenotypic explorations at diagnosis and follow up of a pediatric patient with leukemic phase of ALK+ anaplastic large cell lymphoma (ALCL) by multiparametric flow cytometry (MFC).

**Case:**

An 8‐color MFC combination of antibodies allowed to identify neoplastic cells in concentrations until 0.02% during minimal residual disease (MRD) monitoring. Immunophenotypic shifts occurred in key markers as CD30, CD7, CD2, and CD5, however neoplastic cells were clearly discriminated from normal populations.

**Conclusion:**

MFC can be a useful tool for ALCL diagnosis and MRD monitoring and may support therapeutic decisions.

## 1 INTRODUCTION

The leukemic phase of anaplastic large cell lymphoma (ALCL) is a rare condition restricted to ALK+ forms of the disease.^1^, ^2^ Peripheral blood (PB) and bone marrow (BM) involvement is associated with poor prognosis,^3^ making it essential to evaluate BM samples for treatment response prediction. In this context, multiparametric flow cytometry (MFC) is a well‐established method that may be applied, as its usefulness was demonstrated for diagnostic screening and minimal residual disease (MRD) monitoring of many hematological malignancies.^4^ Here, we describe the utility of MFC for the diagnosis and follow up of a pediatric patient with ALK+ ALCL with leukemic presentation. Further, an 8‐color panel permitted to identify the neoplastic cells, despite some immunophenotypic shifts during the treatment.

## 2 METHODS

### 2.1 Samples

All samples were obtained after receiving written informed consent by the donor's legal representative according to the Declaration of Helsinki. The study was approved by the local ethics committee. BM aspirates were sequentially obtained for MFC analysis at day 0, +22, +48, and +64 of therapy; PB samples were collected at day 0 and +99; and samples from a cervical lymph node and cerebrospinal fluid (CSF) at day 0.

### 2.2 Multiparameter flow cytometry (MFC) studies

EuroFlow SOPs were used for sample preparation, instrument setup, data acquisition and data analysis, as previously described.^5^ More details are described in supplemental material. Monoclonal antibodies panels are available at Table S1.

## 3 CASE REPORT

A 6‐year‐old boy was admitted with fever, weight loss and malaise during the past 40 days, plus cervical and abdominal lymphadenopathy, hepatosplenomegaly and anemia (hemoglobin level of 90 g/L ‐ reference value [RV]: 110–128 g/L). White blood cells (WBC) count was 7520 × 10^9^/L (RV: 5700–9900 × 10^9^/L), platelet count 184 × 10^9^/L (RV: 227–350 × 10^9^/L), LDH of 1312 U/L (RV: 120–246) and C‐reactive protein of 61 mg/L (RV: <10 mg/L). Serological analyses and blood culture were all negative. A cervical lymph node biopsy was performed and histopathological (HP) and immunohistochemical (IHC) analyses showed a ALK+ ALCL (CD30^+^/ALK^+^/EMA^+^/Ki67^+(≥80%)^/CD20^−^/CD3^−^/CD4^−^/EBV^−^/CD68^−^/CD1a^−^). BM biopsy staging by HP + IHC did not show infiltration of neoplastic cells at diagnosis.

**FIGURE 1:**
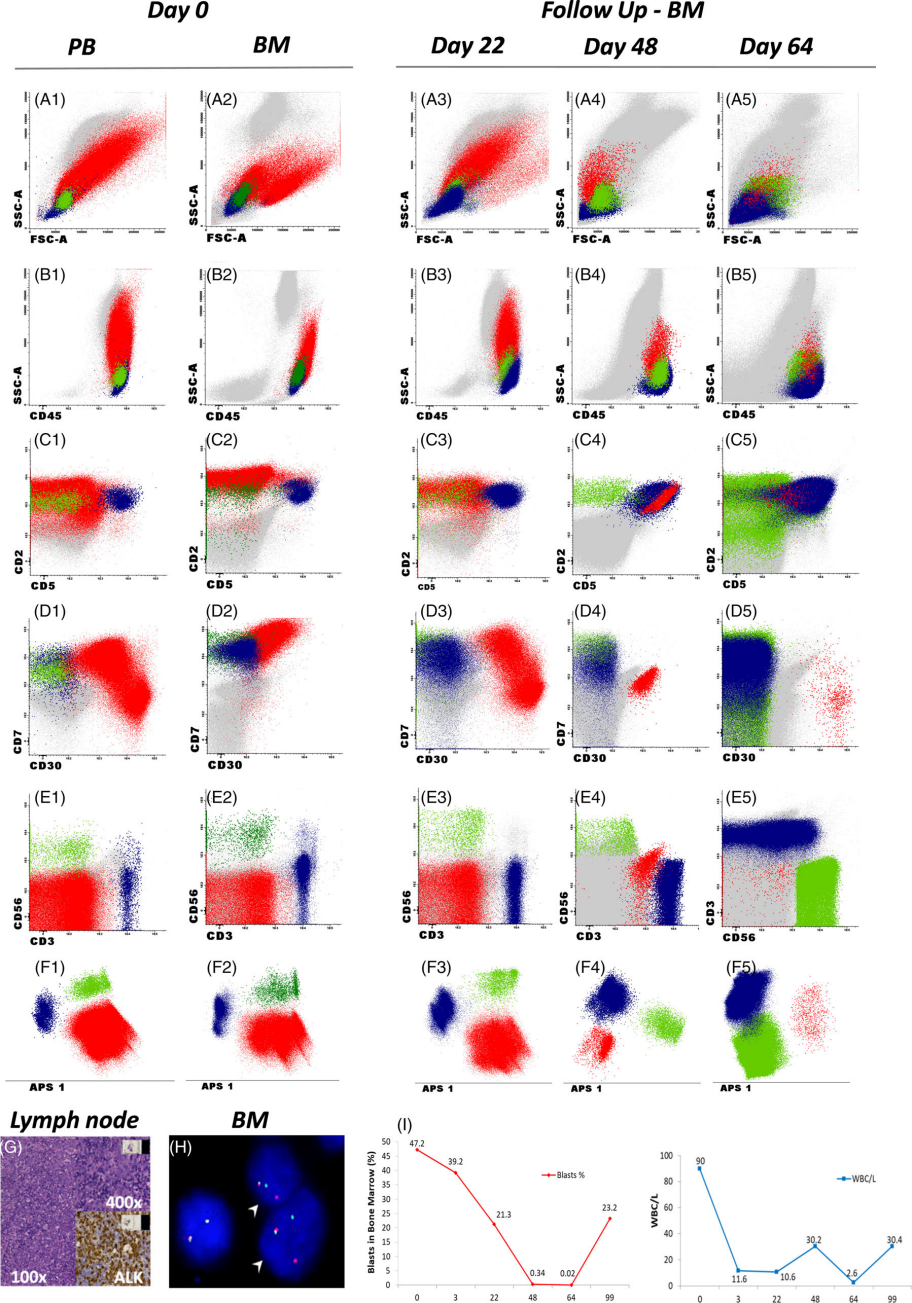
Diagnosis and minimal residual disease monitoring of a patient with ALCL. Flow cytometric immunophenotypic analysis of peripheral blood samples (A1–F1) shows ALCL tumor cells (red) with a high sideward scatter (SSC‐A), high levels of CD45 and CD30 expression, heterogeneous expression of cyCD3, CD2 and CD7 and the absence of surface CD3 and CD56. This phenotype differs from normal T cells (blue), which show: low SSC‐A, CD45+, CD2+, cyCD3+, CD3+, CD7+, and CD30−. NK cells CD56 positive are colored green. Histopathological analysis (G) of the cervical lymph node sample was performed. Paraffin‐embedded tissue sections were immuno‐stained with monoclonal antibodies against CD30, ALK, EMA, Ki67, CD20, CD3, CD4, EBV, CD68 and CD1a; and sections were counterstained in Harris hematoxylin. Image G shows 100× and 400× magnification and ALK staining by immunohistochemistry. FISH analysis (H) of a tumor infiltrated bone marrow sample using dual color ALK break apart probe showing normal and abnormal ALK (2p23) gene arrangement. Fused red/green signals (overlap as a yellow signal) represent the normal ALK gene locus and the separated red and green signals represent an underlying ALK rearrangement (i.e., translocation ‐ arrowhead). Flow cytometric analysis of an infiltrated bone marrow sample at diagnosis (A2–F2) exhibits a similar phenotype of tumor cells found in peripheral blood, except for a positive CD7 and low CD30 expression. Minimal residual disease monitoring in bone marrow samples (A3–F3; A4–F4; A5–F5) shows the presence of ALCL tumor cells in all time points. ALCL cells presented different patterns of CD30 expression during MRD monitoring in comparison to the diagnostic sample. Line graphs (I) show the relationship between white blood cell counts (blue line) and tumor cell percentages in total nucleated cells (red line). Results are shown from the beginning of treatment (Day 0) until the last time point of MRD monitoring (Day +99). Peripheral blood sample collected at Day +99 shows a high number of neoplastic cells, indicating treatment failure

The patient received cytoreductive chemotherapy for 4 days, but his WBC count increased up to 90 × 10^9^/L. In PB, we found 47% of neoplastic cells with an ALCL immunophenotypic profile (Figure 1A1–F1): SSC^int/hi^/FSC^int/hi^/CD30^+(34%)/++(66%)^/CD34^−^/CD45^++^/sCD3^−^/cyCD3^+^/CD7^+/−^/CD2^++^/CD5^−^/CD99^+^/TdT^−^/CD1a^−^/CD4^+lo^/CD8^+/−^/TCRab^−^/TCRgd^−^/CD56^−^/CD45RA^−/+^/CD117^−^/HLADR^+/++^/CD123^+lo/+^/CD44^−/+^/ MPO^−^/CD33^−^/CD13^+lo/+^/CD10^−^/CD19^−^/cyCD19a^−^/CD20^−^. It was possible to identify two different populations of neoplastic cells according to CD30 expression – both positive for this marker, but one with a higher expression (Figure 1D1). On the other hand, CD56 can be a useful target for differentiation of ALCL cells and NK cells (Figure 1E1–E5), as both populations may express CD2 (Figure 1C1–C5) and CD7 (Figure 1D1–D5), for example.

BM immunophenotypic analysis was also compatible with leukemic phase of ALCL: 37% of nucleated cells showed the same immunophenotypic pattern found on PB tumor cells, except for the more intense and homogeneous CD7 and CD2 expression with less intense CD30 labeling (Figures 1A2–F2). FISH analysis on BM showed an ALK+ form of ALCL (Figure 1H). The CSF was not infiltrated and the final diagnosis was stage IV ALK+ anaplastic large cell lymphoma.

The disease was treated according to ALCL 99 (arm IV) protocol, the international protocol for the treatment of childhood ALCL,^6^ being high dose methotrexate (HD‐MTX) omitted from the 1st cycle due to ascites and pleural effusions. Before the 2nd cycle of chemotherapy, MRD evaluation in BM by MCF showed 21.3% of neoplastic cells with a phenotypic profile similar to the one found at diagnosis. MRD decreased to 0.34% at the evaluation performed prior to the 3rd cycle, followed by a reduction to 0.02% prior to the 4th cycle of chemotherapy. In Figure 1, one can note that CD30, CD7, CD5, and CD2 suffered some immunophenotypic shifts during the treatment but neoplastic cells were easily identified using the combination of markers and the principal components analysis (PCA) plot. The population with higher expression of CD30 displayed chemoresistance and this fact can be better observed by comparing dotplot D1 to dotplot D5, both of Figure 1. Dotplot D1 was performed at diagnosis and shows the two populations of ALCL cells according to CD30 and dotplot D5, performed at day 64 of treatment, shows only the population with higher CD30 expression, resistant.

A fully compatible bone marrow donor (10/10) was found for allogenic hematopoietic stem cell transplantation. However, at day 14 of the 4th cycle of chemotherapy, the patient was admitted to the intensive care unit (ICU) due to a multiorgan failure (with negative blood cultures), severe mucositis and ALCL progression (23.2% of tumor in PB, as detected by MFC), and died after 8 days in the ICU.

## 4 DISCUSSION

BM involvement of ALCL is observed in only 10%–30% of cases and the leukemic form of ALK+ ALCL is associated with a poor outcome,^3^ as the case reported here with a very aggressive clinical behavior. Tsuyama et al have pointed the necessity of more sensitive techniques for BM staging in ALCL patients as even with the association of HP and IHC less than half of patients with infiltrated BM were identified.^7^ Thus, MFC appears as a highly sensitive tool for ALCL diagnosis and follow up. In addition, ALCL MRD detection by MFC was less sensitive (10^‐5^), but less expensive and time consuming compared with the MRD study by reverse‐transcriptase polymerase chain reaction (RT‐PCR) technique.^8^ In Table 1, a summary of the MFC studies performed to characterize ALCL cells, mostly at diagnosis, can be found.^2^, ^3^, ^9^, ^10^, ^11^ One of these studies^8^ established an elegant strategy for MRD detection of ALCL using 4‐color MFC more than 10 years ago. Of note, in our case report we used an 8‐color MFC combination of antibodies for ALCL MRD monitoring that together with PCA plots were useful to identify neoplastic cells even when immunophenotypic shifts occurred in such key markers as CD30, CD7, CD2, and CD5. The automatic population separation tool was of great importance for the identification of distinct neoplastic populations upon diagnosis. CD56 (N‐CAM) is frequently expressed on non‐hematopoietic tumor cells, but it can be negative in part of ALCL cases,^2^, ^10^, ^11^ as the present case. By applying PCA, the automatic population separation was able to consider a variety of parameters that can better separate populations and subpopulations of tumor cells. It adds another layer of accuracy to the diagnosis as it provides a multivariate analysis that considers the markers included in the eight colors and forward and side scatter parameters.^5^

**TABLE 1:** Previous reports of the use of MFC for the detection of tumor cells at diagnosis and/or follow up of ALCL

	Number of ALCL cases studied	% Samples with ALCL cells detected by MFC	Panel/Cytometer	Sensitivity of MRD detection	Moment of the study	Immunophenotypic pattern of ALCL cells described by MFC at diagnostic									
						ALK+	CD56	CD45	CD30	CD4	CD2	CD3	CD5	CD7	CD8
Juco et al^9^	19	100% (19/19)	4‐color/FACSCalibur	‐	*Diagnostic*	33.3% (3/9)	NP	100% (19/19)	100% (19/19)	63% (12/19)	71% (12/17)	32% (6/19)	26% (5/19)	32% (6/19)	21% (4/19)
Damm‐Welk et al^8^	^a^		4‐color/FACSCalibur	10^−5^	*Follow up (MRD)* ^a^	ND	ND	ND	ND	ND	ND	ND	ND	ND	ND
Kesler et al^10^	29	86% (25/29)	3‐color/FACScan or 4‐color/FACSCalibur	‐	*Diagnostic*	61.9% (13/21)	60% (6/10)	92% (23/25)	92% (23/25)	80% (20/25)	72% (18/25)	40% (10/25)	32% (8/25)	32% (8/25)	Negative
Muzzafar et al^11^	23	82.65 (19/23)	4‐color/FACSCalibur	‐	*Diagnostic*	57.9% (11/19)	33% (2/6)	100% (19/19)	100% (19/19)	36.4% (4/11)	77.8% (7/9)	47.4% (9/19)	20% (3/15)	50% (5/10)	16.7% (2/12)
Gadage et al^2^	2	100% (2/2)	8‐color/FACSCantoII	‐	*Diagnostic*	100% (2/2)	50% (1/2)	100% (2/2)	100% (2/2)	50% (1/2)	50% (1/2)	Negative	Negative	Negative	Negative
Shen et al^3^	15	86% (13/15)	4‐color/FACSCalibur	‐	*Diagnostic*	100% (13/13)	NP	100% (13/13)	100% (13/13)	84.6% (11/13)	76.9% (10/13)	53.8% (7/13)	38.4% (5/13)	61.5% (8/13)	Negative
Canellas et al. (2021)	1	100% (6/6)	8‐color/FACSCantoII	10^−5^	*Diagnostic & Follow up (MRD)*	Positive	Negative	Positive	Two populations: 1. Low positive 2. High positive	Low positive	Positive	Positive	Negative	Heterogeneous	Heterogeneous

Abbreviations: ALCL, anaplastic large cell lymphoma; MFC, multiparameter flow cytometry; MRD, minimal residual disease; ND, not done; NP, not provided.

^a^ The MRD detection was performed in dilutional experiments by mixing ALCL cell lines with normal human peripheral blood or bone marrow samples.

Immunophenotypic shifts identification can be also potentially useful to guide immunotherapies (e.g., anti‐CD30 therapy with brentuximab), with the follow up of target molecules expression. Despite the CD30 expression described in the majority of ALCL cases presented in the literature,^2^, ^3^, ^9^, ^10^, ^11^ some studies do not describe the CD30 expression intensity. In PB of the case here reported, we found two tumor cell populations according to different CD30 expression patterns at diagnosis, as well as a quite variable CD30 expression pattern at different MRD time points.

Such variability of CD30 expression in our ALCL case could be due to a cleavage of CD30 into a soluble form (sCD30). Further, such variation of CD30 expression could potentially affect MFC sensitivity for MRD detection depending on what antibodies combinations are used. In this report, despite the variation of CD30 expression, we were able to detect low numbers of ALCL tumor cells in PB and BM (10^‐4^) using a multiparametric analysis that employs the 'out‐of‐normal' strategy instead of only searching for tumor cells with an immunophenotype similar to the diagnostic sample. Further, the EuroFlow Bulk Lysis SOP was used to increase the number of cells acquired in the MRD studies by MFC and reach higher levels of sensitivity.

Due to the low frequency of ALCL cases, multicentric studies to design and validate a fully standardized panel of antibodies for MRD monitoring by MFC can be fundamental to disseminate it in routine clinical laboratories.

## CONFLICT OF INTEREST

We would like to declare that we have no conflicts of interest on publishing this manuscript.

## AUTHOR CONTRIBUTIONS

**Maria Clara Canellas:** Conceptualization (lead); investigation (lead); validation (lead); visualization (lead); writing ‐ original draft (lead). **Enrico Bruno‐Riscarolli:** Conceptualization (lead); investigation (lead); validation (lead); visualization (lead); writing ‐ original draft (lead). **Cristiane Ferreira‐Facio:** Conceptualization (lead); investigation (equal); methodology (lead). **Daiana Lopes:** Investigation (supporting); methodology (lead). **Vitor Botafogo:** Writing ‐ original draft (supporting); writing‐review & editing (supporting). **Deborah Sutter:** Investigation (supporting). **Roberia de Pontes:** Investigation (supporting). **Marcelo Land:** Project administration (equal); supervision (equal). **Cristiane Milito:** Investigation (lead); methodology (lead). **Elaine Costa:** Conceptualization (lead); funding acquisition (lead); methodology (lead); project administration (lead); resources (lead); software (lead); supervision (lead).

## ETHICS STATEMENT

The present study was approved by the institutional board review and patient consent was obtained for publication of this report.

## Supporting information


**Appendix S1.** Supporting information.
Gives extra information about methods, including samples, histopathological (HP) and immunohistochemical (IHC) analyses, multiparameter flow cytometry (MFC) studies and multicolor interphasefluorescent in situ hybridization (iFISH).

**TABLE S1.** Gives extra information about monoclonal antibodies combinations and fluorochrome used for the MFC studies.

## Data Availability

The data that support the findings of this study are available from the corresponding author upon reasonable request.
