# Supplementary material for: Immunophenotypic shifts during minimal residual evaluation in a case of leukemic form of anaplastic large cell lymphoma ALK+
Source: Cancer Rep (Hoboken). 2021 Aug 11;5(7):e1526. doi: 10.1002/cnr2.1526 (PMC9327670; doi:10.1002/cnr2.1526)
Supplement: Supplementary file 1 — Appendix S1. Supporting information. Gives extra information about methods, including samples, histopathological (HP) and immunohistochemical (IHC) analyses, multiparameter flow cytometry (MFC) studies and multicolor interphasefluorescent in situ hybridization (iFISH). [file CNR2-5-e1526-s001.docx]

**SUPPLEMENTAL MATERIAL**

**METHODS:**

***Samples.*** At diagnosis, a cervical lymph node sample was obtained by excisional biopsy at the operating room, as well as a BM aspirate and a biopsy of the posterior iliac crest. BM aspirates were sequentially performed for MFC analysis at day 0, +22, +48 and +64 of therapy; PB samples were collected at day 0 and +99.

A cerebrospinal fluid (CSF) sample (1mL) was obtained by lumbar puncture at diagnosis, collected in a tube containing 100μL of Transfix (Cytomark, Buckingham, United Kingdom) and carefully homogenized.

***Histopathological (HP) and Immunohistochemical (IHC) analyses.*** Paraffin-embedded tissue sections were cut and dried overnight at 56°C, dewaxed in xylene and rehydrated using serial concentrations of ethanol. Heat-mediated antigen retrieval was performed. Endogenous peroxidase activity was blocked with 5% alcoholic hydrogen peroxide for 30 minutes. Slides were then incubated overnight with the primary antibodies and, afterwards, with the secondary antibody for 20 minutes. Tissue sections were stained with monoclonal antibodies against CD30, ALK, EMA, Ki67, CD20, CD3, CD4, LMP1, CD68 and CD1a for immunohistochemical studies. Streptavidin–biotin–peroxidase was used for the detection and diaminobenzidine as chromogen. Sections were counterstained in Harris haematoxylin.

***Multiparameter Flow Cytometry (MFC) studies***.

Briefly, staining was performed in 100µL of sample to which the appropriate volume of fluorochrome-conjugated antibody reagents directed against cell surface markers (Supplemental Table S1) was added and then, sample incubation was performed for 30 minutes at room temperature (RT) in the dark. For surface antibodies combination, after cell surface markers stained, 2 mL of FACS lysing solution -Becton/Dickinson Biosciences (BD), San Jose, CA- diluted 1:10 (vol/vol) in distilled water was subsequently added, followed by an incubation for another 10 minutes at RT, and a washing step, after which cells were resuspended in 500 μL of phosphate buffered saline containing 0.2% Bovine Serum Albumin (PBS-BSA). For intracellular plus surface markers combination, after surface membrane staining, the cell suspension was washed and incubated with solution A of the Fix & Perm Reagent Kit (Nordic-MUbio, Susteren, The Netherlands) for 15 minutes at RT. Afterward, washed cells were incubated for another 15 minutes at RT with solution B of the Fix & Perm Reagent Kit and antibodies against the intracellular markers. Stained cells were washed once and resuspended in 500 μL of PBS-BSA.

Samples used for MRD evaluation were submitted to the EuroFlow BulkLysis standard operating procedure prior to staining with monoclonal antibodies, as previously described (6). Further, the CSF sample was washed three times with PBS-BSA 0.2% before the staining step. In all cases, cells were acquired immediately after sample preparation was completed or stored at 4°C for a maximum of 1 hour until acquisition in the flow cytometer. All EuroFlow SOPs are freely available in full at www.EuroFlow.org. A minimum of 1 x 105 cell events was acquired for diagnostic samples, 5 x 10^6^ events for MRD evaluation and the whole sample (4x10^4^ events) for CSF study, using a FACSCanto II Flow Cytometer - 8-color/3-lasers (BD Biosciences, San Jose, CA) and the FACSDiva software (BD Biosciences). For data analysis, Infinicyt software (Cytognos, Salamanca, Spain) was used.

***Multicolor interphasefluorescent in situ hybridization (iFISH).*** Multicolor interphase FISH (iFISH) studies were performed on an aliquot of the same single cell suspension prepared from BM infiltrated with tumor cells after fixation in 3/1 methanol/acetic (v/v) (Merck) using dual color ALK break apart probe (Vysis Inc). Fixed cells were dropped into cleaned slides that were then sequentially incubated with a solution containing 0.1 mg/ml pepsin (Sigma) (10 minutes at 37°C), fixed in 1% acid-free formaldehyde (Merck) (10 minutes at RT) and de-hydrated in increasing concentrations of etanol (Merck) in water (70%, 95%, 100%). Once dried, the slides containing both the cells’ DNA and the probes’ DNA were denatured at 78°C (10 minute) and subsequently hybridized overnight at 37°C. After this incubation, slides were sequentially washed for 5 minutes at 46°C in 50% formamide (Biosolve) in 2X SSC (Sigma) and then in 2X SSC. Afterward, cells were counter-stained with DAPI (Sigma) and mounting medium Vectashield (Vector Laboratories Inc, Burlingame, CA) were used. Hybridization spots per nuclei were quantified in a BX51 ﬂuorescence microscope (Olympus, Hamburg, Germany) with 100× oil objective. At least 100-200 nuclei were evaluated per slide. The break-apart signal pattern (1R1G1F, one red, one green, and one fusion signal) was quantified.
