# Supplementary material for: Immunophenotypic shifts during minimal residual evaluation in a case of leukemic form of anaplastic large cell lymphoma ALK+
Source: Cancer Rep (Hoboken). 2021 Aug 11;5(7):e1526. doi: 10.1002/cnr2.1526 (PMC9327670; doi:10.1002/cnr2.1526)
Supplement: Supplementary file 2 — TABLE S1. Gives extra information about monoclonal antibodies combinations and fluorochrome used for the MFC studies. [file CNR2-5-e1526-s002.docx]

**SUPPLEMENTAL TABLE S1** Monoclonal antibodies combinations and fluorochrome used for the MFC studies.

| Fluorocromes | PacB | PacO | FITC | PE | PerCP-Cy5.5 | PE-Cy7 | APC | APC-H7 |
| --- | --- | --- | --- | --- | --- | --- | --- | --- |
| Antibody combination used on BM and PB samples | cyCD3 | CD45 | CD2 | CD30 | CD5 | CD56 | CD7 | CD3 |
|  | UCHT1 | HI30 | RPA-2.10 | BerH8 | L17F12 | N901/NKH1 | 124-1D1 | SK7 |
|  | BD  (San Jose, CA) | Exbio  (Vestec, Czech Republic) | BD  (San Jose, CA) | BD  (San Jose, CA) | BD  (San Jose, CA) | Beckman Coulter  (Brea, CA) | eBioscience  (San Diego, CA) | BD  (San Jose, CA) |
| Antibody combination used on CSF sample | CD20 | CD45 | CD2 | CD30 | CD5 | CD56 | CD7 | CD3+CD14 |
|  | 2H7 | HI30 | RPA-2.10 | BerH8 | L17F12 | N901/NKH1 | 124-1D1 | SK7 / MφP9 |
|  | Exbio  (Vestec, Czech Republic) | Exbio  (Vestec, Czech Republic) | BD  (San Jose, CA) | BD  (San Jose, CA) | BD  (San Jose, CA) | Beckman Coulter (Brea, CA) | eBioscience  (San Diego, CA) | BD  (San Jose, CA) |

BM: bone marrow; PB: peripheral blood; CSF: cerebrospinal fluid; PacB: Pacific Blue; PacO: Pacific Orange; FITC: Fluorescein Isothiocyanate; PE: Phycoerythrin; PerCP-Cy5.5: Peridin Clorophyll Protein Cyanine5.5; PE-Cy7: Phycoerythrin Cyanine7; APC: Allophycocyanin; APC-H7: Allophycocyanin Hilite7.
